# Targeting the Midgut Secreted PpChit1 Reduces *Leishmania major* Development in Its Natural Vector, the Sand Fly *Phlebotomus papatasi*

**DOI:** 10.1371/journal.pntd.0000901

**Published:** 2010-11-30

**Authors:** Iliano V. Coutinho-Abreu, Narinder K. Sharma, Maricela Robles-Murguia, Marcelo Ramalho-Ortigao

**Affiliations:** Department of Entomology, Kansas State University, Manhattan, Kansas, United States of America; National Institutes of Health, United States of America

## Abstract

**Background:**

During its developmental cycle within the sand fly vector, *Leishmania* must survive an early proteolytic attack, escape the peritrophic matrix, and then adhere to the midgut epithelia in order to prevent excretion with remnants of the blood meal. These three steps are critical for the establishment of an infection within the vector and are linked to interactions controlling species-specific vector competence. PpChit1 is a midgut-specific chitinase from *Phlebotomus papatasi* presumably involved in maturation and degradation of the peritrophic matrix. Sand fly midgut chitinases, such as PpChit1, whether acting independently or in a synergistic manner with *Leishmania*-secreted chitinase, possibly play a role in the *Leishmania* escape from the endoperitrophic space. Thus, we predicted that silencing of sand fly chitinase will lead to reduction or elimination of *Leishmania* within the gut of the sand fly vector.

**Methodology/Principal Findings:**

We used injection of dsRNA to induce knock down of *PpChit1* transcripts (dsPpChit1) and assessed the effect on protein levels post blood meal (PBM) and on *Leishmania major* development within *P. papatasi*. Injection of dsPpChit1 led to a significant reduction of *PpChit1* transcripts from 24 hours to 96 hours PBM. More importantly, dsPpChit1 led to a significant reduction in protein levels and in the number of *Le. major* present in the midgut of infected *P. papatasi* following a infective blood meal.

**Conclusion/Significance:**

Our data supports targeting PpChit1 as a potential transmission blocking vaccine candidate against leishmaniasis.

## Author Summary

For a successful development within the midgut of the sand fly vector, *Leishmania* must overcome several barriers which are imposed by the vector. The ability to overcome these barriers has been associated with species specificity, and interference with the sand fly vector-parasite balance can change the outcome of the infection in the vector. Recently, our group has carried out a transcriptome assessment of the sand fly *Phlebotomus papatasi* midgut, uncovering many transcripts possibly associated with the barrier to *Leishmania* development. In order to validate the role of such genes, we have developed a dedicated RNA interference (RNAi) platform to assess whether RNAi targeting such genes can reduce *Leishmania major* development. PpChit1 is a midgut-specific chitinase presumably involved in the maturation/degradation of the peritrophic matrix in the gut of the sand fly after a blood meal. Our results show that knockdown of *PpChit1* via RNAi led to a significant reduction of *Le. major* within the gut, supporting the potential use of PpChit1 as a target for transmission blocking strategies against sand fly-transmitted leishmaniasis.

## Introduction

Emerging and reemerging vector-borne diseases pose significant threats to human and animal health [1]. The emergence of insecticide resistance as well as the lack of other efficient insecticidal tools to control disease vectors imply that new methodologies need to be developed in order to reduce vector-borne disease transmission [1]. For this, the study of vector-pathogen interaction pinpointing factors underlying vector competence can reveal new molecular targets to be disrupted, preventing pathogen transmission [2], [3].

In sand flies, midgut molecules are known or believed to be involved in defining a species ability to transmit *Leishmania* in nature. For a successful development within the midgut of the sand fly vector, *Leishmania* must overcome several barriers that include an early proteolytic attack [4], [5], [6], [7], [8], the need to escape the peritrophic matrix (PM) [8], [9], [10], [11], [12], and attachment to the midgut epithelia to prevent excretion with the remnants of the blood meal [13], [14], [15], [16].

Attachment to midgut epithelia has long been associated with the type of lipophosphoglycan (LPG) present on the surface of *Leishmania*, and is associated with defining sand fly-*Leishmania* pairs in nature [15], [16], [17]. For *Leishmania major* V1 strain, with LPG displaying highly decorated side chains with prominent galactose residues, we demonstrated that PpGalec, a *P. papatasi* galactose-binding protein, is the docking site for *Le. major* on the midgut epithelium of *Phlebotomus papatasi* [14]. Recently, LPG-independent midgut binding has been associated with the degree of glycosylation detected on proteins expressed by midgut epithelial cells [18].

For events leading up to the midgut binding, such as early parasite survival during the proteolytic attack and escape of the endoperitrophic space, some investigators suggested that midgut proteases, such as trypsins and chymotrypsins, also are responsible for defining vector-*Leishmania* specificity [4], [5], [6], [7]. Such proteases were shown to be specially harmful to transitional stages amastigotes [8].

A role of the PM on sand fly vector competence was suggested through comparisons of *Leishmania* development in different sand fly species displaying different PM degradation rates [9], [10], [11]. Studies later revealed a dual role for the sand fly PM in parasite development; protecting *Leishmania* from digestive enzymes in the beginning of blood digestion, yet becoming a barrier to parasite escape when mature [8]. Recent data also indicate that an anterior PM plug located at the junction between the anterior and posterior midgut acts as a barrier to *Leishmania* migration towards the stomodeal valve [12].

Regarding *Leishmania* escape from the PM, it was firstly proposed to be solely accomplished by a parasite chitinase [19]. Further work demonstrated that a *Le. mexicana* chitinase-overexpressing strain had an accelerated escape from the PM in *Lutzomyia longipalpis* [20]. However, since the characterization of a blood induced chitinolytic system in the sand fly midgut [21], it became apparent that the parasite must take advantage of the sand fly peak chitinolytic activity within midgut, approximately 40–48 hours after a blood meal, for their escape [12], [21].

PpChit1 is presumably involved in PM maturation/degradation in *P. papatasi* [8]. Based on the fact that *Leishmania* must escape the PM, and that this escape may be aided by the vector's own chitinase, we predicted that *PpChit1* knock down (via RNAi) would interfere with *Le. major* development. Our data indicates that dsRNA-mediated silencing of *PpChit1* transcripts leads to a reduction in the parasite load within the midgut of *P. papatasi*, pointing to the role of this molecule in *P. papatasi* vector competence and its potential for the development of a transmission-blocking vaccine.

## Methods

### Ethics statement

The use of animals during this study was reviewed and approved by the Kansas State University Institutional Animal Care and Use Committee (KSU-IACUC).

### Sand fly rearing, dissection, and infection with *Le. major*

*P. papatasi* (Israeli strain -PPIS) was reared in the Department of Entomology, Kansas State University, according to conditions described [21]. For all experiments, three-to-five day old female sand flies were used. Blood feeding was performed through a chicken skin membrane attached to a feeding device. Prior to sand fly feeding, fresh mouse blood was heat inactivated for 30min at 56°C and supplemented with 50 µl/ml of Pen/Strep solution (MP Biomedicals, Solon, OH, USA) as well as 1 mM ATP (MP Biomedicals). Sixteen to twenty four hours after blood feeding, fully engorged females were separated from partially engorged and non-blood fed by anesthetizing flies with CO_2_ and examining the midgut distension under a stereoscope microscope. Only fully fed individuals were maintained for further analyses.

Fully engorged sand fly midguts were individually dissected on RNAse free (cleaned with ELIMINase, Fisher Scientific, Pittsburgh, PA, USA) glass slides, transferred to 50 µl of 1× PBS buffer (RNase free, pH 7.4; Fisher Scientific), and thoroughly homogenized using a hand held tissue homogenizer and RNAse-free pestle. Half the homogenate volume (25 µl) was transferred to 350 µl of RLT buffer (supplemented with 1% β-mercaptoethanol) provided by the RNA extraction kit (RNAeasy mini kit, Qiagen, Valencia, CA, USA) and stored at −80°C for quantitative real-time PCR assays. The remaining 25 µl of midgut homogenate was used in Western blot assays, as described below.

Infections of sand flies with *Le. major* amastigotes V1 strain were done by addition of 5×10^6^ parasites/ml into the blood meal. *Le. major* amastigotes were harvested from BALB/c mouse footpads lesions formed roughly 30 days after inoculation with 5×10^5^ parasites from late phase culture according to [22].

### dsRNA synthesis and injection

dsRNA for *PpChit1* were synthesized using the primers PpChit1/T7i_2–F (5′–TAATACGACTCACTATAGGGAGAATGAAGATATCATTGTGTGC-3′) and PpChit1/T7i_2–R (5′– TAATACGACTCACTTAGGGAGATCAGCATTGGACCAGGAAGG-3′), which contain the complete T7 promoter and amplify the full length sequence encoding the mature PpChit1. PCR reactions were performed with 0.5pmoles of each primer along with 1 µl of cDNA (synthesized from midgut dissected at 72 h post-blood meal, PBM), and 10 µl of GoTaq PCR master mix (Promega, Madison, WI, USA). The 20 µl PCR reactions were carried according to the conditions: 10 cycles of 95°C for 1 min, 55°C for 1 min, and 72°C for 1 min and 15 sec, followed by 35 cycles 95°C for 1 min, 65°C for 1 min, and 72°C for 1 min and 15 sec. The reaction products were purified and concentrated using the YM-100 filters (Millipore, Billerica, MA, USA), and 1 µg DNA was used for dsRNA synthesis using the Megascript RNAi kit (Ambion, Austin, TX, USA). dsRNA synthesis reactions were performed for four hours at 37°C, and the products were further purified following manufacturer's recommendations. Thereafter, dsRNAs were suspended in ultra-pure water and further purified and concentrated to approximately 3.5 mg/ml or 4.5 mg/ml using the YM-100 filters (Millipore). The positive control provided by the Megascript RNAi kit (Ambion; used in Real-Time PCR and Western blot assays) or a dsRNA specific to a green fluorescence protein gene (dsGFP [23]; for parasite counting assays) was used as controls for dsRNA injection assays.

For dsRNA injections, individual females were anesthetized with CO_2_, kept on a cold dish, and injected intra-thoraxically with either 23 nl (3.5 mg/ml, 80.5 ng) or 32 nl (4.5 mg/ml, 144 ng) of dsRNA using Nanoject II microinjector (Broomall, PA, USA). Immediately following injection, flies were transferred to a 500 ml plastic container, provided with 30% sugar embedded cotton, and maintained inside a high humidity chamber (85–95% humidity at 25°C). Flies were allowed to recover for 48 hours and blood fed on an uninfected blood meal through a chicken membrane, as described above.

### RNA isolation and cDNA synthesis

Total RNA was isolated from individual midguts dissected as described above. RNA extraction was carried out using the RNAeasy mini kit (Qiagen) following manufacturer's instructions. Following extraction, the Turbo DNA-*free* kit (Ambion) was used to eliminate DNA contamination. After quantification, 25 ng total RNA was used for cDNA synthesis using 200 units of SuperScript III Reverse Transcriptase (200 u/µl), 2.5 µM Oligo (dT)_20_ primer, and 0.5 µM dNTPs (10 mM). These reagents were incubated at 65°C for 5 minutes (min) and kept in ice for at least 1 min. This step was followed by addition of a mix containing 4 µl 5× SuperScript III Reverse Transcriptase First-Strand Buffer, 5 mM DTT (0.1 M), 20 Units of RNaseOUT to the reaction. The mixture was incubated for one hour at 50°C and stored at −20°C. All the reagents for cDNA synthesis were purchase from Invitrogen (Carlsbad, CA, USA).

### Quantitative real-time PCR analyses

Real-Time PCR reactions were performed using BioRad SYBR green and BioRad iCycler (BioRad, Hercules, CA, USA). The reactions were carried out in duplicate using 0.5 µl cDNA, 6pmoles of each primer (10 µM), 10 µl of 2× SYBR green, and 8.3 µl of Ultra Pure DNase/RNase-Free Water (Invitrogen). The primers used for chitinase amplification were PpChit_137F (5′ - ATGATCTGCATGGTTCTTGG - 3′) and PpChit_137R (5′ - GGAGCTCCATTTCGAATCC - 3′) while the S3 primers (Pp40S_S3_136F: 5′ - GGACAGAAATCATCATCATG – 3′ and Pp40S_S3_136R: 5′ – CCTTTTCAGCGTACAGCTC – 3′) were used for amplifications of the housekeeping control gene (encoding the protein S3 of ribosomal subunit 40S). The reaction cycle of 94°C for 1 min, 57°C for 1 min, and 72°C for 30 sec was repeated 40 times, and the amplification profiles were assessed using the BioRad iCycler software (BioRad).

### PpChit1 anti-sera and Western blot analyses

Polyclonal anti-PpChit1 sera were obtained by injecting three month old female BALB/c mice subcutaneously into the ears. Mice were injected three times in two weeks intervals with approximately 10 µg of purified VR2001 plasmid [24] encoding the mature chitinase protein [21] per mouse ear. Blood was collected from the submandibular vein (“cheek bleed”) of injected animals and antibody levels accessed via Easy-Titer IgG Assay Kit (Pierce, Rockford, IL, USA). Sera were maintained at −20°C until used. For Western blots, seven midgut extracts from flies injected with dsPpChit1 and dsControl were pooled together in RNasefree microcentrifuge tubes containing 1 µl of complete protease inhibitor (Thermo Scientific, Rockford, IL, USA) and concentrated using the YM-10 filters (Millipore). Total protein concentration in midgut extracts was quantified using BCA Protein Assay Kit (Thermo Scientific). Similar proteins amounts (5 µg per lane) from midguts of dsPpChit1 and dsControl injected sand flies were fractionated on 10% Bis-Tris NuPAGE gels (Invitrogen). Proteins were transferred to a nitrocellulose filter (Whatman, Dassel, Germany), incubated with PpChit1 antisera (1:100 dilution) overnight at 4°C, washed three times in TBS-T (1× TBS buffer with 0.1% tween-20) for 15 minutes each time. Blot was incubated with anti-mouse conjugated to alkaline phosphatase (1:10,000 in TBS-T) antibodies (Promega) for one hour at room temperature and washed in TBS-T as indicated above. The protein bands (56 kDa, [21]) were visualized using the Western Blue substrate for Alkaline Phosphatase (Promega). Alternatively, Western blot was incubated with anti-mouse-Horseradish Peroxidase secondary antibody (1:10,000) and detected with SuperSignal West Pico Chemiluminescence Substrate (Thermo Scientific) in chemiluminescence assays. Densitometry analysis was performed using the TotalLab TL100 software (Nonlinear Dynamics, Durham, NC, USA).

### *P. papatasi* dissection and parasite counting

In order to assess the *PpChit1* knockdown effects on *Le. major* development, 80.5 ng of dsRNA was injected intra-thoraxically into *P. papatasi*, and flies were fed on an infected blood meal as described above. Midguts from fully engorged-only flies were dissected at 48 h and 120 h after the infective blood meal and homogenized in 30 µl of PBS buffer (pH 7.4). Parasites were counted with a hemocytometer. Two independent experiments were carried out for each time point.

### Statistical analysis

Mann-Whitney U test was performed to compare expression profiles as well as parasite numbers between sand flies injected with either dsRNA targeting *PpChit1* transcripts (dsPpChit1) or the dsRNA control (dsControl) injected flies. D'Agostino & Pearson omnibus normality test was performed to assess whether parasite numbers followed a normal distribution. The Chi-square test (or Fisher's exact test) was performed in order to assess whether dsPpChit1-injected flies exhibit altered *Le. major* load compared to the dsControl-injected flies. Parasite infection load in flies dissected at 48 h post infection was scored according to parasite numbers in the sand fly midgut as no parasite, light infection (1–1,000 parasites), moderate infection (1,001–10,000), or heavy infection (>10,000), in accordance to [25]. For flies dissected at 120 h PBM parasite loads were categorized in two groups: zero or light infections (0–1,000 parasites) was arranged as one group, and moderate infection (>1,000 parasites) as another. Differences were considered statistically significant at p<0.05, and tests were carried out using GraphPad Prism v. 5.01 software (GraphPad Software, Inc).

## Results

### dsPpChit1 effects on mRNA levels

**Figure 1:**
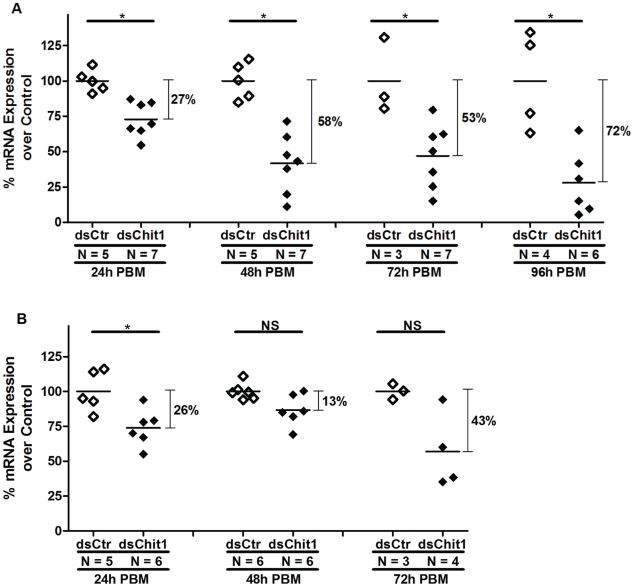
dsRNA effect on *PpChit1* RNA levels. Real-Time PCR comparing the mRNA level of *PpChit1* between flies injected with 80.5 ng (A) or 144 ng (B) of dsPpChit1 (dsChit1) or dsControl (dsCtr) double-strand RNAs. Significant *PpChit1* transcript reduction was exhibited by dsPpChit1 injected flies at 24 h (A and B), 48 h, 72 h, and 96 h PBM (A). *PpChit1* mRNA levels were normalized with the S3 housekeeping gene. Results are presented as a percent of *PpChit1* expression levels in dsPpChit1 injected flies over the mean of *PpChit1* expression levels in dsControl injected flies (considered as 100%) for each time point. The variance in PpChit1 expression in dsControl injected flies is also shown. Each dot represents *PpChit1* RNA levels in a single fly. Horizontal bars indicate mean expression level. *: Statistically significant at p<0.05.

Injection of 80.5 ng of dsRNA into the sand fly thorax targeting the midgut expressed *PpChit1* gene led to a significant decrease in *PpChit1* mRNA levels in comparison with the control dsRNA-injected flies (Figure 1). Reduction of *PpChit1* expression after a blood meal varied over time. Twenty four hours after blood meal (and 72 h after injection of dsPpChit1), a 27% reduction of *PpChit1* transcripts was detected (Figure 1A). At 48 h PBM (previously shown to be the maximum activity for PpChit1 [21]) and at 72 h PBM, reductions of 58% and 53% on average of the *PpChit1* expression were observed (Figure 1A). Finally, at 96 h PBM (120 h after dsRNA injection), when no chitinolytic activity was detected [21], the reduction in *PpChit1* expression was 72%.

On the other hand, injection of 144 ng of dsPpChit1 into *P. papatasi* thorax displayed a weaker reduction in *PpChit1* expression levels than injection of 80.5 ng (Figure 1B). Although similar expression reduction at 24 h PBM was exhibited (26%, Figure 1B), expression differences between dsPpChit1 and dsControl injected flies at 48 h and 72 h PBM were lower (13% and 43%, respectively) than detected at the same time points when 80.5 ng of dsRNA was injected (Figure 1B). These differences could be occurring due to a still obscure feedback loop for transcription activation upon knock down, as proposed elsewhere [26].

### dsPpChit1 effects on protein levels

**Figure 2:**
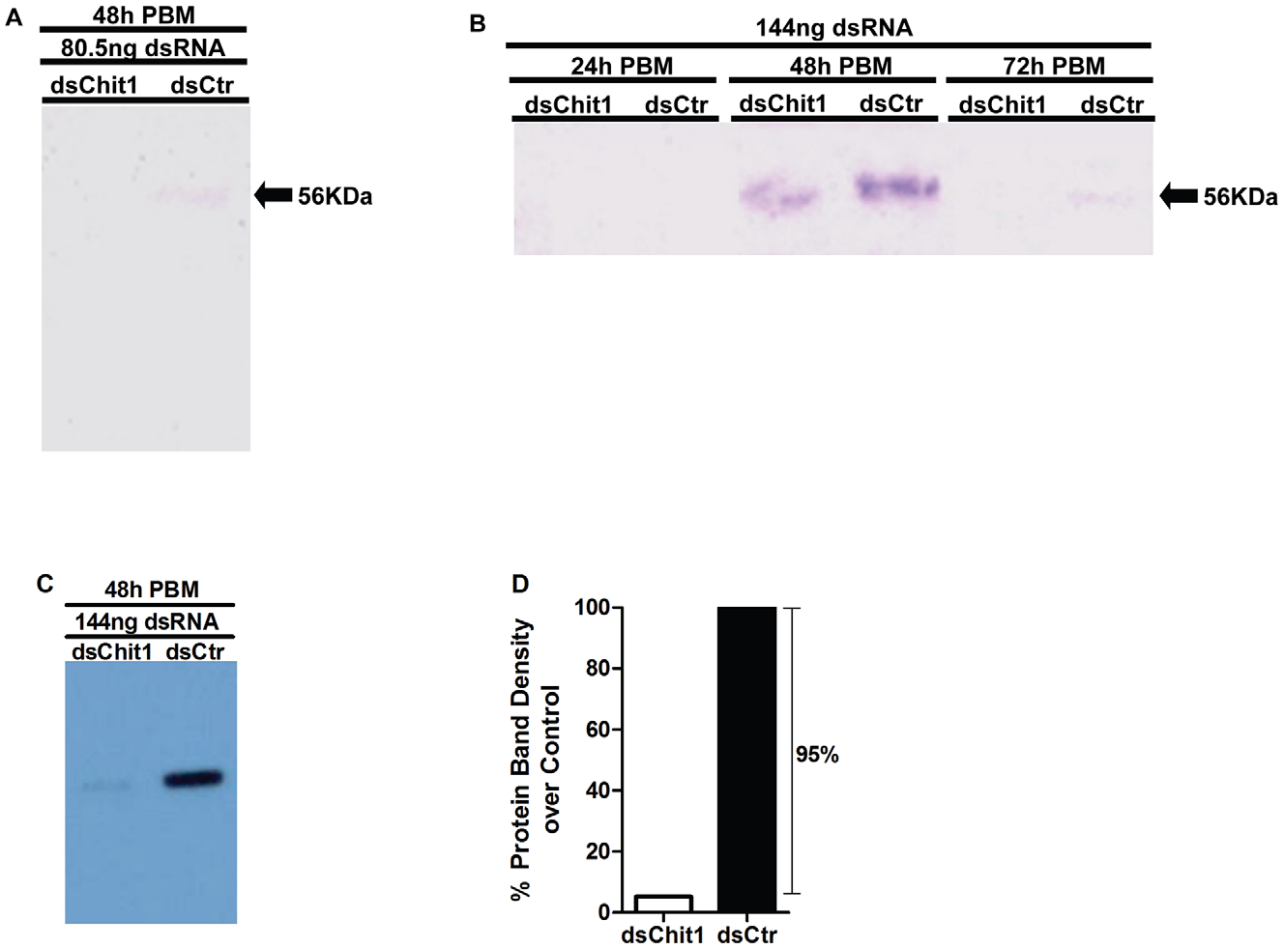
dsRNA effect on PpChit1 protein levels. (A). Western blot assay pointing to *PpChit1* knock down in dsPpChit1 injected flies (80.5 ng dsRNA) at 48 h PBM. (B). Midgut extracts from flies injected with 144 ng dsPpChit1 (dsChit1) displayed weaker bands (56 kDa) than dsControl (dsCtr) injected flies at 48 h and 72 h PBM. A–B, Colorimetric development. (C). Western blot assay depicting strong PpChit1 expression reduction in flies injected with 144 ng dsPpChit1 (dsChit1) compared with dsCtr injected ones at 48 h PBM (Chemiluminescence development). (D). Densitometry analysis of PpChit1 protein bands obtained in the chemiluminescence assay revealing 95% reduction in PpChit1 expression between dsPpChit1 and dsControl injected flies.

Silencing of the *PpChit1* message RNA produced a concomitant reduction in the amount of PpChit1 protein as determined by Western blots (Figure 2). Similar to the Real-Time PCR data, reduction in PpChit1 protein levels in dsPpChit1 injected flies was detected at 48 h and 72 h PBM (Figures 2A–C) when either 80.5 ng or 144 ng of dsRNA was injected. No PpChit1 expression was detected at 24 h PBM (Figure 2B). Likewise, densitometry analysis of blot developed using a chemiluminescence method displayed 95% reduction in PpChit1 protein levels at 48 h PBM when 144 ng dsPpChit1 (Figure 2C and D). Interestingly, the corresponding time point only led to 13% reduction of PpChit1 mRNA levels, as shown in Figure 1B.

### dsPpChit1 effects on *Le. major* development within *P. papatasi* midgut

**Figure 3:**
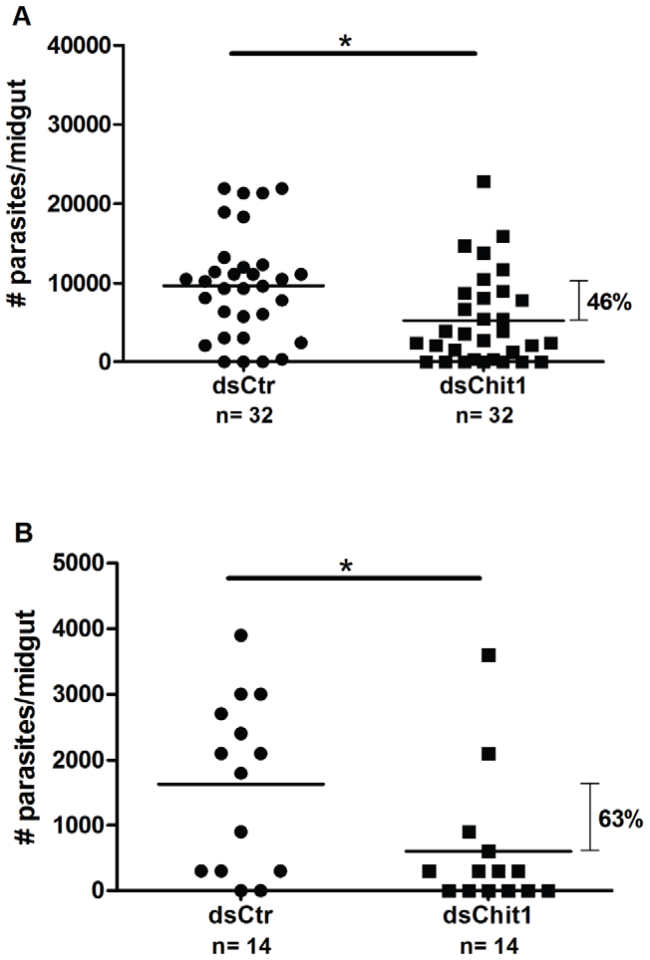
dsRNA effect on *Le. major* development. Intra-thoracic injections of dsPpChit1 (80.5 ng) reduce *Le. major* load in *P. papatasi* midgut. (A). At 48 h PBM, *Le. major* density was reduced on average 46% in dsPpChit1 (dsChit1) injected compared with dsControl (dsCtr) injected. (B). *Le. major* parasites per midgut were further reduced at 120 h PBM in dsPpChit1 injected flies, reaching on average 63% reduction over the dsControl injected ones. Each dot represents parasite number in a single *P. papatasi* midgut. Horizontal bars display mean parasite numbers. n: Number of flies analyzed. *: Statistically significant at p<0.05. Graphs represent one similar result of two independent experiments.

As injection of either 80.5 ng or 144 ng of dsRNA targeting *PpChit1* transcripts are capable of significantly reducing *PpChit1* expression levels in the midgut of *P. papatasi* (Figure 1 and 2), we assessed the effects of injecting 80.5 ng of the dsRNA on *Le. major* development within the injected flies. Following the injection of the PpChit1 dsRNA, flies were provided an infective blood meal, and dissected at different time points after feeding. Our results demonstrate that dsPpChit1-targeted knock-down resulted in significant reductions in parasite load within the sand fly midgut as the numbers of *Le. major* were reduced by 46% (or 1.85 fold) at 48 h post infection (Figure 3A) and by 63% (or 2.70 fold) at 120 h PBM post infection (Figure 3B).

The injection of dsPpChit1 also affected the range of parasite loads. An analysis of the range of parasite load at 48 h and 120 h post infection points to a normal distribution of parasite numbers in the dsControl-injected flies (48 h PBM, p = 0.51, and 120 h PBM, p = 0.26, D'Agostino & Pearson omnibus normality test), whereas for dsPpChit1-injected flies this distribution was significantly affected (48 h PBM, p = 0.004, and 120 h PBM, p<0.0001, D'Agostino & Pearson omnibus normality test).

**Figure 4:**
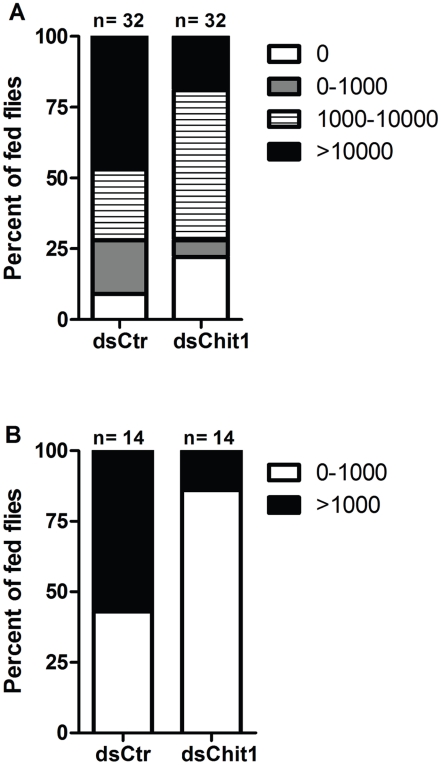
Effect of dsRNA injection on *Le. major* infection level in *P. papatasi*. Parasite load was categorized according to the number of *Le. major* per midgut. (A) Percentage of sand flies injected with either dsCtr or dsChit1 exhibiting no infection (0 parasites), as well as light (1–1,000 parasites), moderate (1,000–10,000 parasites), or heavy (>10,000 parasites) infection at 48 h PBM. Differences are statistically significant (Chi-square, p = 0.01). (B) Percentage of sand flies injected with either dsCtr or dsChit1 exhibiting either no parasites or light infection (0–1000 parasites), or moderate infection (>1,000 parasites) at 120 h PBM. Differences are statistically significant (Fisher's exact test, p = 0.04). n: Number of flies dissected.

Changes in *P. papatasi* infection levels following silencing of PpChit1 were further confirmed by comparing infection prevalence. For instance, injection of dsPpChit1 reduced the prevalence of heavy infection from 47% (dsControl-injected) to 19%, and of light infection from 19% (dsControl-injected) to 6% at 48 h post blood feeding (Figure 4A). Likewise, moderate infections levels were reduced from 57% (dsControl-injected) to 14% at 120 h post infection (Figure 4B).

## Discussion

After a blood meal, sand flies synthesize a PM type 1 that is fully developed at approximately 36–40 h PBM [27]. In addition to compartmentalizing the blood meal and protecting the epithelia, the sand fly PM serves an additional dual role regarding *Leishmania* infection: as a barrier to these parasites but also as protection against proteolytic attack on transitional-stage amastigotes [8], [20], [28], [29], [30]. In order to successfully complete its cycle within the sand fly, *Leishmania* nectomonads must escape from endoperitrophic space, through the PM, to prevent being passed together with remnants of the digested blood meal [8].

We have previously characterized a functional, blood-induced chitinolytic system, in the midgut of *P. papatasi* and *L. longipalpis* sand flies [21], [31]. We also demonstrated that polyclonal antibodies to PpChit1 inhibit the midgut chitinolytic activity *in vitro*, and this effect also was shown across different sand fly species [21]. PpChit1 is presumably involved in the maturation and degradation of *P. papatasi* PM (as is its ortholog in *L. longipalpis*, LlChit1) [21], [31], and addition of allosamidin, a chitinase inhibitor to the infective blood meal of this sand fly led to entrapment of *Le. major* within the peritrophic space [8]. Although allosamidin may have also inhibited chitinase secreted by *Leishmania*, taken together, these data suggested that PpChit1 also can be involved with *Leishmania* escape from the endoperitrophic space.

To address whether silencing of *PpChit1* transcripts via RNAi-induced pathway would affect *Le. major* development within its natural vector, *P. papatasi*, we synthesized a dsRNA specifically targeting *PpChit1*.

Injection of dsRNA targeting specific transcripts has now been widely applied in disease vectors and proven an invaluable tool for the understanding of underlying events in pathogen-vector relationships [32], [33], [34]. In sand flies, gene silencing with dsRNA was first applied to *L. longipalpis* cell culture [35], inducing a non-specific antiviral response. Recently, dsRNA injection of adult sand flies led to a specific reduction of Xanthine dehydrogenase expression [36], and to an effect on *Le. mexicana* development when a midgut trypsin produced by *L. longipalpis* was silenced [30].

The midgut chitinase PpChit1 is only expressed following a blood meal [21]. Thus, following injection of dsPpChit1 double-stranded RNA, sand flies were blood fed and midguts dissected at different intervals after feeding. Specific silencing of *PpChit1* transcripts was detected by quantitative real-time PCR analyses (Figure 1), with concomitant knock down of PpChit1 protein levels assessed by Western blots (Figure 2).

Based on the presumptive role of PpChit1 in the maturation and degradation of the PM1, we expected that silencing of this gene would lead to entrapment of *Leishmania* within the endoperitrophic space. Our results are consistent with this hypothesis, as *Le. major* load was reduced 120 h PBM in midguts of dsPpChit1 injected *P. papatasi* (Figures 3 and 4) suggesting that PpChit1 is indeed involved in PM1 degradation. Moreover, reduction of the *Le. major* load at 48 h PBM in dsChit1 compared to control-injected flies might have been caused by at least two scenarios: 1) a reduction in nutrient availability in the endoperitrophic space as the PM may be less permeable to proteolytic enzymes, or in the contrary, 2) to inability of parasites to escape leading to longer exposure to digestive enzymes inside the peritrophic space. Regardless of the mechanism, it still remains to be determined.

Future studies will assess whether this is a feasible approach in preventing transmission from an infected animal to a naïve host. Moreover, the results support the targeting of PpChit1 as a mean to interfere with *Leishmania* development within the sand fly – a candidate transmission-blocking vaccine.
